# Antibacterial Activity and Mechanism of Lacidophilin From *Lactobacillus pentosus* Against *Staphylococcus aureus* and *Escherichia coli*

**DOI:** 10.3389/fmicb.2020.582349

**Published:** 2020-10-29

**Authors:** Yinglian Zhu, Shuang Zhang

**Affiliations:** College of Food Science and Engineering, Qingdao Agricultural University, Qingdao, China

**Keywords:** *Lactobacillus pentosus*, lacidophilin, *Staphylococcus aureus*, *Escherichia coli*, antibacterial mechanism

## Abstract

The main purpose of this study was to explore the antibacterial activity and mechanism of lacidophilin from *Lactobacillus pentosus* against *Staphylococcus aureus* and *Escherichia coli*. The effects of temperature, enzyme, metal ions, and pH on the antibacterial activity of *L. pentosus* were evaluated. The result showed that lacidophilin had good thermal stability and could be decomposed by trypsin completely. The antibacterial ability was affected by high concentration of metal ions, and the best antibacterial ability was acquired under acidic conditions. The antibacterial mechanism of lacidophilin was explored through studying cytomembrane injury, phosphorus metabolism, protein changes, and oxidative stress response of the indicator bacteria. It was shown that lacidophilin destroyed the cytomembrane of the bacteria and increased the cytomembrane permeability, which resulted in the leak of proteins, nucleic acids, and electrolytes. In addition, it further restrained phosphorus metabolism, caused changes of some protein contents, and increased cytomembrane lipid peroxidation and cell oxidative damage. All these might inhibit the growth of bacteria and even cause their death. This study identified a natural biological preservative with strong antibacterial activity against both Gram-positive and Gram-negative foodborne pathogens. The high antibacterial activity against the two types of bacteria reflected its potential in food preservation used as a natural food preservative.

## Introduction

The food industry has made significant progress in food standards, food processing, and food testing, but health and safety concerns still persist. It was estimated that about one-third of food is wasted due to spoilage each year (Gills et al., 2015). Some physical and chemical methods have been adopted to extend the shelf life of foods, among which, the addition of chemical preservatives is the most effective way. However, long-term intake of chemical preservatives has adverse effects on human health (Yang et al., 2014). Therefore, more attention has been paid to natural preservatives for their safe and non-polluting character. Bacteriocins have been widely used in foods as natural preservatives because of their wide sources and diverse structures, which have high efficiency in killing various foodborne pathogens and spoilage bacteria (Lv et al., 2018). Lacidophilin is a kind of bacteriocin produced by lactic acid bacteria (LAB), which has advantages than those from other biological resources because LAB is generally recognized as safe (GRAS) microorganisms (Sequeiros et al., 2015). However, most lacidophilin can only inhibit affinis strains of LAB, which limits their applications. Nisin, currently being used widely in food preservation, mainly inhibits most Gram-positive bacteria and spore bacteria and has no antibacterial effect on Gram-negative bacteria. Therefore, more researchers are exploring lacidophilin with wide range antibacterial spectrum. Bifidocin A, produced by *Bifidobacterium animalis* BB04, has been reported by Liu et al. (2017), and the antibacterial activity of bifidocin A against *Listeria* was mainly achieved by destroying the integrity of the cell membrane and increasing membrane permeability. A novel lacidophilin produced by *Lactobacillus plantarum* against *Pseudomonas fluorescens* has been reported by Lv et al. (2018), and it demonstrated wide antimicrobial spectrum against fish pathogens and spoilage bacteria. Previous studies have indicated that bacteriocins are generally antibacterial proteins or peptides (Lv et al., 2018; Vasilchenko et al., 2018). However, the number of bacteriocins is far less than the number of antimicrobial peptides developed (Vasilchenko et al., 2018). In view of the advantageous characteristics of lacidophilin, the development of lacidophilin with high antimicrobial activity against the two types of bacteria is urgent.

In recent years, probiotics have become a hot research topic, due to their safety, nutritional and health value, and antimicrobial function. *Lactobacillus pentosus* (*L. pentosus*) is a Gram-positive lactobacillus, which is widely used in the processing of fermented meat products (Liu et al., 2008; Zhu et al., 2019). The lacidophilin of LAB was considered to be critical for sausage processing because it enhanced the quality and safety of the sausages (Zhu et al., 2020). However, there are only a few studies that focused on the lacidophilin produced by *L. pentosus*. Pentocin 31-1 was identified as anti-*Listeria* lacidophilin produced by *L. pentosus* 31-1 (Liu et al., 2008). Motahari et al. (2016) optimized the fermentation conditions of lacidophilin by *L. pentosus* and revealed that low concentration of sodium chloride promoted the production of lacidophilin. The study by Hurtado et al. (2011) indicated that the appropriate concentration of sodium chloride could improve the bacteriostatic activity of *L. pentosus* B96 lacidophilin, compared with non-saline control and high-concentration sodium chloride group. However, few studies focused on the lacidophilin produced by *L. pentosus* against the two types of bacteria and explored its antibacterial mechanism.

*Escherichia coli* (*E. coli*) is one of the most common Gram-negative foodborne pathogens, which usually was used and is an indicator bacterium in test for fecal contamination of food. It can cause diarrhea, gastroenteritis, and a series of complications (Miri et al., 2017). *Staphylococcus aureus* (*S. aureus*) is a common Gram-positive foodborne pathogen that contaminates various foods, and staphylococcal foodborne diseases from food contamination pathogen, and is regarded as the second most common foodborne illnesses in the world (Di Pinto et al., 2005). The focus of this study is on the antimicrobial activity of *L. pentosus* lacidophilin against *S. aureus* and *E. coli* and further explore its antibacterial mechanism. The fermentation supernatant of *L. pentosus* (lactic acid interference excluded) was used as the crude extract of lacidophilin. The effects of heat, enzyme, metal ions, and pH on the antibacterial effect of lacidophilin were measured to evaluate its antibacterial activity. In addition, the antibacterial mechanism of lacidophilin was further explored through studying cytomembrane injury, phosphorus metabolism, protein changes, and oxidative stress response of the indicator.

## Materials and Methods

### Strains

*Lactobacillus pentosus* (strain no. 22226) and *L. plantarum* (strain no. 23941) were provided by China Center of Industrial Culture Collection (CICC). *E. coli* (strain no. 1.8723) and *S. aureus* (strain no. 1.8721) were provided by China General Microbiological Culture Collection Center (CGMCC).

### Main Reagents

Fluorescein diacetate (FDA), propionyl iodide (PI), sodium dodecyl sulfate, acrylamide, and methylenebisacrylamide were provided by Suo Lai Bao Biotechnology Co., Ltd. Pepsin, papain, trypsin, and amylase were provided by Sinopharm Group Chemical Reagent Co., Ltd., Malondialdehyde (MDA), superoxide dismutase (SOD), peroxidase (POD), and catalase (CAT) reagent test kits were provided by Nanjing Jiancheng Biological Engineering Research Institute.

### Cell-Free Supernatant Containing Lacidophilin Preparation

*Lactobacillus pentosus* was picked from MRS slope with an inoculation loop, transferred to 10 ml of MRS broth, and incubated at 37°C for 24 h. Then, 10 ml of the inoculum was transferred to 100 ml of MRS broth and incubated at 37°C for 72 h. Subsequently, the inoculum (OD_600_ = 2.350) was centrifuged at 8,000 × *g* for 15 min at 4°C. The supernatant was collected, and pH was adjusted with NaOH (1 mol/L) to 5.5 to eliminate the interference of organic acids. Thus, the *L. pentosus* cell-free supernatant containing lacidophilin (*L. p.* supernatant) was obtained, which was used for subsequent antibacterial activity measurement.

### Antibacterial Activity Measurement

The antibacterial activity of *L. pentosus* lacidophilin was carried out utilizing the agar diffusion method according to the previous report (Cai et al., 2019) with minor modifications: MRS medium (10 ml) containing 2% agar was used as the bottom plate. MRS medium (7 ml) containing 0.7% agar and 5% indicator bacteria cell suspension (OD_600_, 0.6) was poured on the bottom plate as the upper plate. After the upper plate was completely solidified, three Oxford cups were placed on the plate. Then, 0.1 ml of the *L. p.* supernatant was injected into the Oxford cup, and the diameter of inhibition zone around the Oxford cup was measured to determine antibacterial activity after being incubated at 37°C for 24 h. The MRS broth (without *L. pentosus* inoculation) was used as the negative control. In addition, the positive control was prepared through substituting *L. pentosus* with the *L. plantarum* strain capable of producing lacidophilin.

### Total Protein Content of the *L. p.* Supernatant

The total protein content of the *L. p.* supernatant was detected with the Coomassie Brilliant Blue G-250 dye method according to the report by Cai et al. (2019). The *L. p.* supernatant (1 ml) was mixed with 5 ml of Coomassie Brilliant Blue G-250 dye and laid for 2 min. The total protein content of the *L. p.* supernatant was evaluated by measuring the OD value of the mixture at 595 nm. The standard curve of protein content was drawn as follows: the standard protein solution (0.0, 0.1, 0.2, 0.3, 0.4, and 0.5 ml) was added to 1 ml of volumetric flasks, respectively, and then the volume was made up to 1 ml with distilled water. Subsequently, 1 ml of the protein solution was mixed with 5 ml of Coomassie Brilliant Blue G-250, respectively, and placed for 2 min. Protein content was evaluated by measuring OD value at 595 nm.

### Effects of Heat, Enzyme, and Metal Ions on Antibacterial Activity

The antibacterial activity was carried out according to the above chapter (section “Antibacterial Activity Measurement”). To determine the thermal stability of lacidophilin, the *L. p.* supernatant was treated at 65, 85, and 100°C in a water bath for 10 min, respectively, and then 0.1 ml of the supernatant was taken out for antibacterial activity measurement. The *L. p.* supernatant without heat treatment was used as the control. To evaluate the effect of enzymes, four enzymes (pepsin, papain, amylase, and trypsin) were added in the *L. p.* supernatant, respectively, to make the final concentration to be 1 mg/ml. After being reacted at 37°C for 2.5 h at appropriate pH, they were heated at 100°C in a water bath for 10 min to inactivate the enzymes (Lv et al., 2018), and then 0.1 ml of the supernatant was taken out for antibacterial activity measurement. The *L. p.* supernatant without enzyme treatment was used as the control. The effect of metal ions was carried out with different concentrations of NaCl, KCl, MgCl_2_, and CaCl_2_ solutions. Different volumes (1, 2, and 4 ml) of sterile metal ions solution (0.1 mol/L, with *L. p.* supernatant as solvent) were added to 4 ml of the *L. p.* supernatant to make the final metal ions concentrations be 0.02, 0.033, and 0.05 mol/L, which corresponded to 1, 2, and 4 ml, respectively. Then, 0.1 ml of the supernatant was taken out for antibacterial activity measurement. The *L. p.* supernatant without metal ions treatment was used as the control.

### Effect of Lacidophilin on the Dynamic Growth of the Two Indicator Bacteria

The indicator bacteria were inoculated in 10 ml of LB broth, incubated at 37°C for 24 h, then transferred to 100 ml of LB broth, and incubated at 37°C for 12 h. After being centrifuged at 8,000 × *g* for 15 min at 4°C, the bacteria cell was resuspended in LB broth to adjust the absorbance value to 0.6 at 600 nm. Then, the bacterial suspension (100 ml) was mixed with an equal volume of *L. p.* supernatant and incubated at 37°C for 1.5, 3.5, 5.0, 7.0, 9.0, and 11.0 h, respectively. The absorbance at 600 nm (Yi et al., 2016) was recorded to measure the effect of lacidophilin on the dynamic growth of the two indicator bacteria.

### Effect of Lacidophilin on Cell Membrane Integrity

#### Fluorescence Spectrum Analysis

The *L. p.* supernatant was prepared according to the above chapter (section “Cell-Free Supernatant Containing Lacidophilin Preparation”). Then, the indicator bacterial suspension (OD_600_, 0.6) (4 ml) was mixed with an equal volume of *L. p.* supernatant and incubated at 37°C for 12 h. The inoculum (5 ml) was taken out and centrifuged at 8,000 × *g* for 15 min at 4°C. Then, the indicator bacteria cells were collected and washed with phosphate buffer solution (PBS, 0.1 mol/L, pH 7.0) three times. The bacterial cells were resuspended in 920 μl of 0.85% NaCl solution, and then 20 μl of FDA (5 mg/ml) and 60 μl of PI (1 mg/ml) were added. After staining at 4°C for 6 h in the dark (Liu et al., 2017), the fluorescence scan spectrum was measured using a fluorescence spectrophotometer at an excitation wavelength of 450 nm.

#### Fluorescence Microscope Observation

The staining steps of the indicator bacteria were consistent with fluorescence spectrum analysis. Then, the stained cells were centrifuged at 8,000 × *g* for 15 min at 4°C, and the cells were collected and washed with PBS (0.1 mol/L, pH 7.0) three times. Finally, the cells were resuspended in 1 ml of PBS (0.1 mol/L, pH 7.0) and observed under a fluorescence microscope.

### Effect of Lacidophilin on Cell Membrane Permeability

Electrolyte leakage of the indicator bacteria was measured according to the method by Shen et al. (2015) with minor modification. The indicator bacteria were suspended in 50 ml of LB broth to make an OD_600_ of 0.6. The bacterial suspension (50 ml) was mixed with an equal volume of *L. p.* supernatant and incubated at 37°C for 2.5 h. The conductivity of the mixture was measured at 0, 0.5, 1.0, 1.5, and 2.5 h, respectively, with a conductivity meter. Before measuring conductivity, 5 ml of the mixture was removed and filtered with 0.45 μm sterile filters to remove bacteria cells.

Leakage of the soluble nucleic acids was detected according to the method by Cai et al. (2019) with minor modification. The indicator bacterial suspension (20 ml, OD_600_, 0.9) was mixed with an equal volume of *L. p.* supernatant and incubated at 37°C for 0, 1.5, 3, 5, and 7 h, respectively. Then, 5 ml of the inoculum was taken out and centrifuged at 10,000 × *g* for 10 min at 4°C, and the supernatant was collected, respectively. Subsequently, 4 ml of the supernatant was taken out to measure the OD value at 260 nm to determine the nucleic acid leakage.

Leakage of the soluble protein was detected with the Coomassie Brilliant Blue G-250 dye method according to the description of the previous chapter (Total Protein Content of the *L. p.* Supernatant). After leakage of the soluble nucleic acids being measured, the remaining supernatant (1 ml) was mixed with 5 ml of Coomassie Brilliant Blue G-250 dye and laid for 2 min. The soluble protein leakage was evaluated by measuring the OD value of the mixture at 595 nm. The standard curve of protein content was drawn as the description of the previous chapter (Total Protein Content of the *L. p.* Supernatant).

### Effect of Lacidophilin on Phosphorus Metabolism

Phosphorus metabolism of the indicator bacteria was measured according to the previous method (Wang et al., 2020) with some modifications. The indicator bacteria were suspended in glucose solution (1 mg/ml) to make an OD_600_ of 0.9. The bacterial suspension (7.5 ml) was mixed with an equal volume of *L. p.* supernatant, and then 1.5 ml phosphorus standard solution (50 μg/ml) was added. After being incubated at 37°C for 0, 0.5, 1.0, 1.5, and 2.5 h, the inoculum (2 ml) was taken out and centrifuged at 8,000 × *g* for 15 min at 4°C, and then the supernatant was collected. A 10% ascorbic acid solution (1 ml) was added in the collected supernatant and stood for 30 s; subsequently, 2 ml of 2.5% molybdate solution was added and reacted for 15 min. The OD value was measured at 700 nm to confirm the phosphorus concentration. The MRS medium (7.5 ml) was used to substitute the *L. p.* supernatant as the blank control to remove the interference of the medium. The standard curve of phosphorus content was drawn as follows: the phosphorus standard solution (0.0, 0.05, 0.1, 0.3, 0.5, 1.0, and 1.5 ml) was added to seven volumetric flasks (25 ml), and then the solution was made up to 25 ml with distilled water. Subsequently, the solution (2 ml) was taken out, mixed with 1 ml of 10% ascorbic acid solution, and stood for 30 s. Finally, 2 ml of 2.5% molybdate solution was added and reacted for 15 min.

### Effect of Lacidophilin on Bacterial Protein

SDS-PAGE was carried out to measure the bacteria cell protein changes according to the method by Cai et al. (2019) with some modifications. The indicator bacterial suspension (4 ml, OD_600_, 0.6) was mixed with an equal volume of *L. p.* supernatant and incubated at 37°C for 18 h. Then, 5 ml of the inoculum was taken out and centrifuged at 8,000 × *g* for 15 min at 4°C to collect the precipitate. After being washed three times with PBS (0.1 mol/L, pH 7.0), the bacteria cells were collected. The collected cells were resuspended in 500 μl of 0.85% NaCl, and then 20 μl of the suspension was taken out, mixed with 10 μl of protein loading buffer, boiled in a water bath for 5 min, and cooled to room temperature instantly. After being centrifuged at 3000 × *g* for 5 min, 15 μl of the supernatant was analyzed with SDS-PAGE gel electrophoresis using 5% concentrated gel and 12% separation gel. The gel was dyed with Coomassie Brilliant Blue R-250 for 30 min and decolorized overnight until the background became transparent.

### Effect of Lacidophilin on Intracellular Enzyme (SOD, POD, and CAT) Activity and MDA Content

The SOD, POD, and CAT activities and MDA content were measured according to the previous method (Jia et al., 2014) with some modifications. The indicator bacterial suspension (10 ml, OD_600_, 0.9) was mixed with an equal volume of *L. p.* supernatant and incubated at 37°C for 5 h. Then, 5 ml of the inoculum was taken out and centrifuged at 8,000 × *g* for 15 min at 4°C to collect the precipitate. After being washed three times with PBS (0.1 mol/L, pH 7.0), the bacteria cells were collected. After being grinded with quartz sand and a mortar in an ice-bath for 5 min, the cells were removed into 10 ml of 0.85% NaCl solution, well mixed, and centrifuged at 8,000 × *g* for 15 min at 4°C. The collected supernatant was used to measure the activity of intracellular enzyme and the content of MDA. The measurements were conducted with SOD, POD, CAT, and MDA kits and read in a microplate reader.

### Statistical Analysis

Statistical analysis was implemented using SPSS version 18.0, and the data were analyzed through multiple comparisons with the Duncan method. A probability level of 0.05 was regarded as a significance limit.

## Results

### Antibacterial Activity of Lacidophilin

No antibacterial effect against *E. coli* and *S. aureus* was observed in the negative control (MRS medium alone) (Table 1), which eliminated the interference of the medium to the antibacterial effect. In addition, adjustment of pH to 5.5 with NaOH (1 mol/L) eliminated the interference of organic acids. The total protein content of the *L. p.* supernatant was 964 μg/ml measured with the Coomassie Brilliant Blue G-250 dye method. The diameters of inhibition zones around the Oxford cup against *E. coli* and *S. aureus* were 19.83 and 18.46, respectively (Table 1), which indicated that the lacidophilin from *L. pentosus* had high activity against the two strains. The cell density of the inoculum had no significant difference (*P* > 0.05) compared with the positive control, but the inhibition effect was higher (*P* < 0.05) than the positive control, which showed that lacidophilin had great potential as a food preservative in food preservation.

**TABLE 1:** Antibacterial activity of lacidophilin.

Groups	OD_600_ (37°C 72 h fermentation)	Inhibition zone diameter (against *E. coli*)	Inhibition zone diameter (against *S. aureus*)
Negative control	0.001 ± 0.001^b^	0 ± 0^c^	0 ± 0^c^
Positive control	2.268 ± 0.189^a^	16.593 ± 0.668^b^	15.257 ± 0.386^b^
The treated group	2.350 ± 0.063^a^	19.830 ± 0.960^a^	18.460 ± 0.685^a^

*Values are represented as mean ± SD from triplicate experiments. Different lowercase superscripts in the same column indicate that the antibacterial effect is significantly different (*P* < 0.05). The diameter of the inhibition zone around the Oxford cup is used to denote the antibacterial activity of lacidophilin.*

### Effects of Heat, Enzyme, and Metal Ions on Antibacterial Activity

**FIGURE 1:**
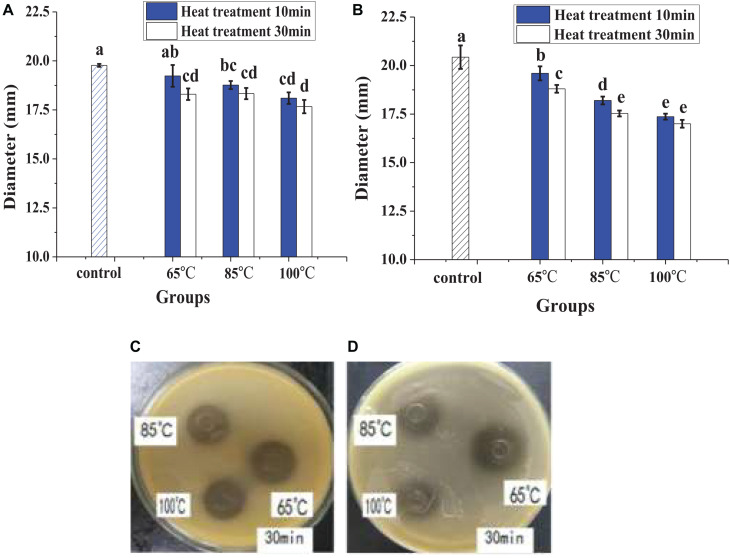
The effects of heat on antibacterial activity, diameter (mm) refers to the diameter of the inhibition zone around the Oxford cup, which displayed the antibacterial activity, and the description of the diameter also applied to the following figure. The bactericidal effect against *E. coli* **(A)** and against *S. aureus* **(B)**, in which different lowercase letters meant that the bactericidal effect was significantly different (*P* < 0.05). The photographs of heat treatment effects on antibacterial activity against *E. coli* **(C)** and against *S. aureus* **(D)** at different temperatures for 30 min.

**FIGURE 2:**
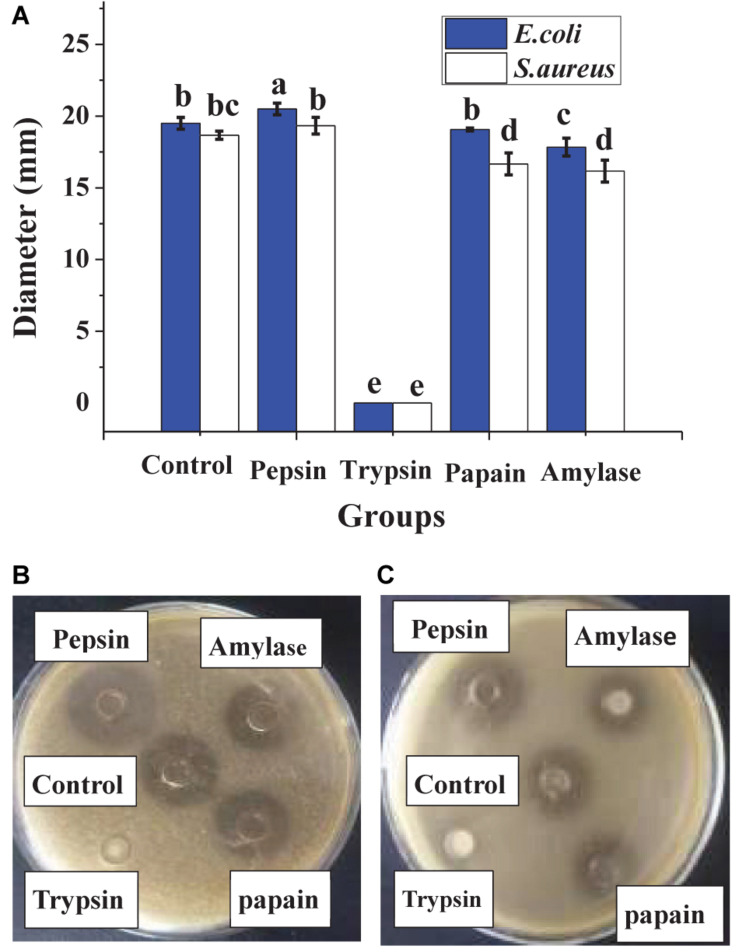
The effects of enzymes on antibacterial activity. The bactericidal effect against *E. coli* and *S. aureus* **(A)**, in which different lowercase letters meant that the bactericidal effect was significantly different (*P* < 0.05). The photographs of enzyme treatment on antibacterial activity against *E. coli* **(B)** and against *S. aureus* **(C)**.

**FIGURE 3:**
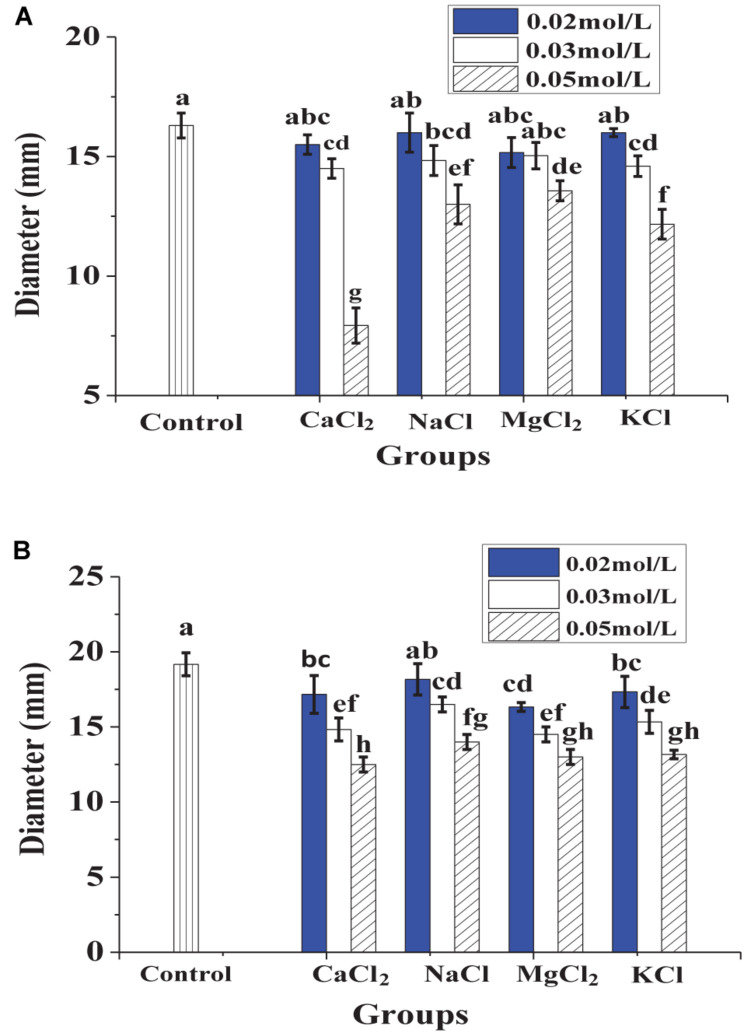
The effects of metal ions on antibacterial activity against *E. coli* **(A)** and *S. aureus* **(B)**, in which different lowercase letters meant that the antibacterial effect was significantly different (*P* < 0.05).

In Figure 1, heat treatment showed a certain negative effect on the antibacterial effect of lacidophilin, and the negative effect increased with heating temperature increased and time prolonged. However, after being treated at 65°C for 30 min, lacidophilin retained about 90% of the antibacterial activity against the two indicator bacteria. After being treated at 85 and 100°C for 30 min, lacidophilin retained about more than 80% of the antibacterial activity against the two bacteria. Figure 2 shows that different enzymes had different effects on the antibacterial effect of lacidophilin. Pepsin had no significant effect on the antibacterial activity (*P* > 0.05). However, the antibacterial effects of lacidophilin against the two bacteria were both weakened after being treated with papain and amylase. After being treated with trypsin, lacidophilin lost its antibacterial effect, which indicated that lacidophilin could be degraded completely by trypsin. In addition, it could be seen from Figure 3 that different types and different concentrations of metal ions had different effects on the antibacterial ability of lacidophilin. As the concentration of metal ions increased, the antibacterial effect against the two indicator bacteria gradually weakened.

### Dynamic Growth Curve of Two Indicator Bacteria

**FIGURE 4:**
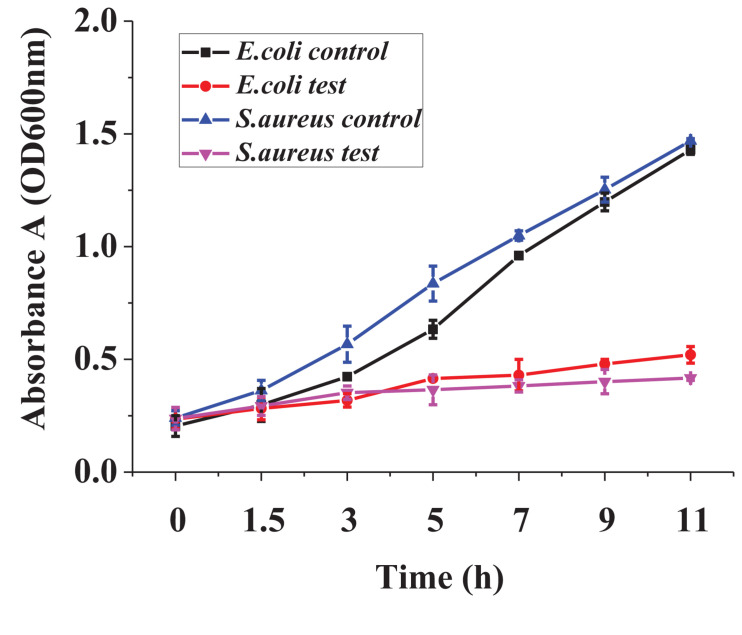
The dynamic growth curve of the two indicator bacteria.

In Figure 4, the dynamic growth of the treated bacteria and the control was similar in the lagged period. After 1.5 h, the difference between treated bacteria and the control increased significantly. The control had a high-speed growth trend, whereas the growth of the treated bacteria was significantly inhibited.

### Effect of Lacidophilin on Cytomembrane Integrity

**FIGURE 5:**
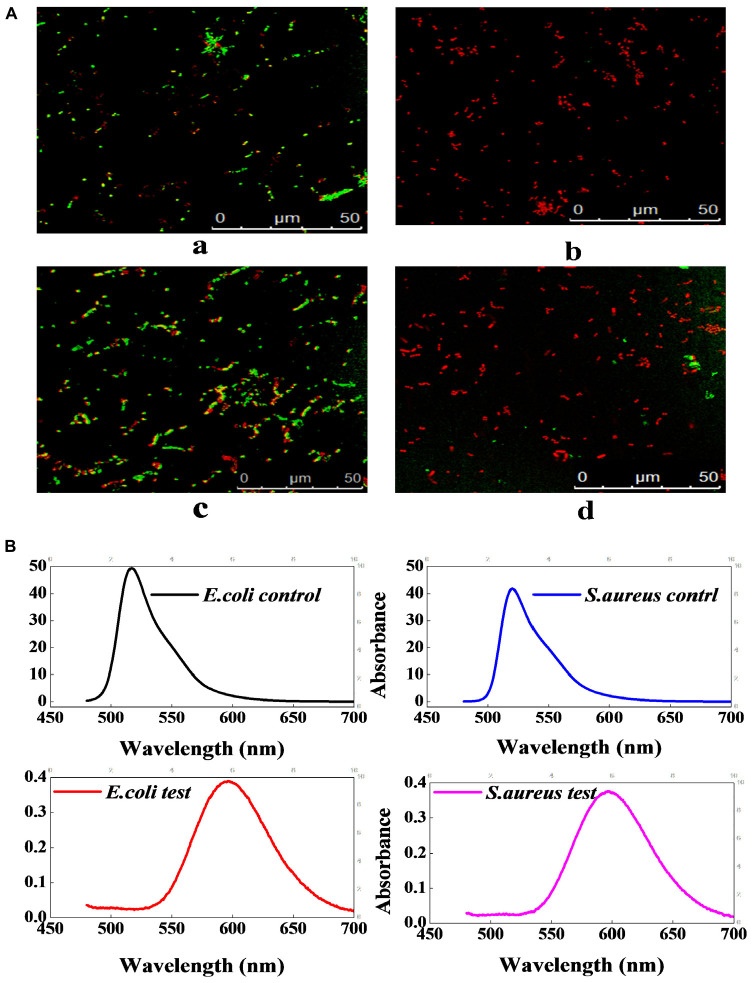
The effect of lacidophilin on the cell membrane integrity of the two indicator bacteria. Fluorescence micrograph of *E. coli* and *S. aureus* with FDA/PI staining **(A)**; the *S. aureus* used as control **(a)**, the treated *S. aureus* **(b)**, the *E. coli* used as control **(c)**, the treated *E. coli* **(d)**. Fluorescence spectrum of *E. coli* and *S. aureus* with FDA/PI staining **(B)**; the excitation wavelength was 450 nm.

Figure 5A shows that the untreated *E. coli* and *S. aureus* emitted green fluorescence, whereas the treated *E. coli* and *S. aureus* both emitted red fluorescence, which illustrated that the cytomembrane integrity of the indicator bacteria had been destroyed by lacidophilin. In the fluorescence spectrum of Figure 5B, the absorption peaks at about 520 nm were both observed in the untreated *E. coli* and *S. aureus*, which was corresponding to the absorbance peak of FDA, whereas the peaks of the treated *E. coli* and *S. aureus* were both moved to about 596 nm, which was consistent with the absorbance peak of PI. The result of the fluorescence spectrum further indicated that the cytomembrane had been destroyed.

### Effect of Lacidophilin on Cytomembrane Permeability

**FIGURE 6:**
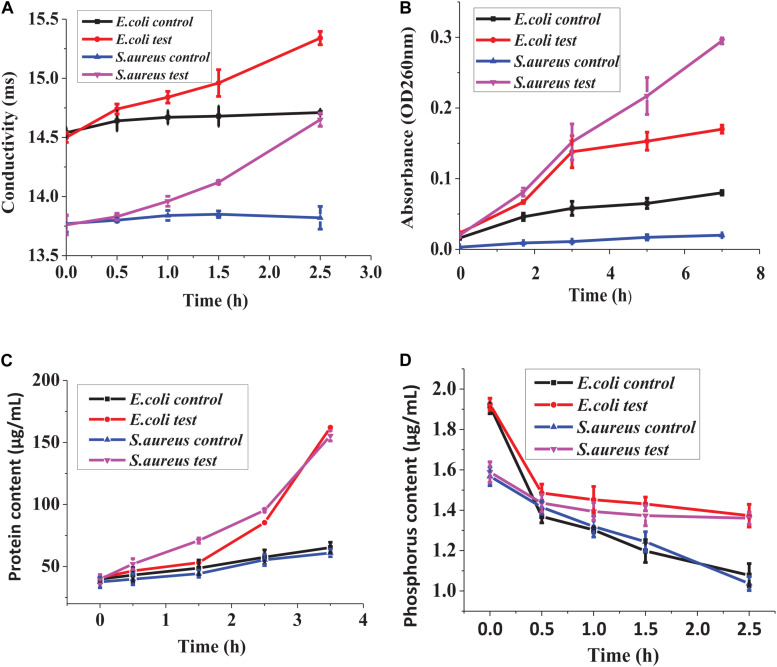
Effect of lacidophilin on cell membrane permeability, leakage of electrolyte **(A)**, leakage of nucleic acid **(B)**, leakage of proteins **(C)**, phosphorus metabolism **(D)**.

Figure 6A shows that the inoculum conductivity of the treated *E. coli* and *S. aureus* increased with the time prolonged, which indicated that the leakage of electrolyte increased, whereas the conductivity of the control did not change significantly. In Figure 6B, the inoculum absorbance of the treated *E. coli* and *S. aureus* at 260 nm was higher than that of the controls, indicating the leakage of nucleic acid from the treated strains. In addition, the absorbance differences between the treated strains and the controls increased significantly with time extension, which showed that the nucleic acid leakage was increasing gradually. In Figure 6C, *S. aureus* showed protein leakage after being treated for about 0.5 h; *E. coli* showed protein leakage after being treated for about 1.5 h. As time went on, protein leakage both kept increasing. In addition, as could be seen from Figure 6D, the phosphorus contents in the inoculum of the treated bacteria and the controls were all decreased with time prolonged. However, the phosphorus contents in the inoculum of the two treated bacteria were both higher than those of the two controls throughout the processing, which indicated that lacidophilin inhibited the phosphorus metabolism of the indicator bacteria.

### Effect of Lacidophilin on Bacterial Protein

**FIGURE 7:**
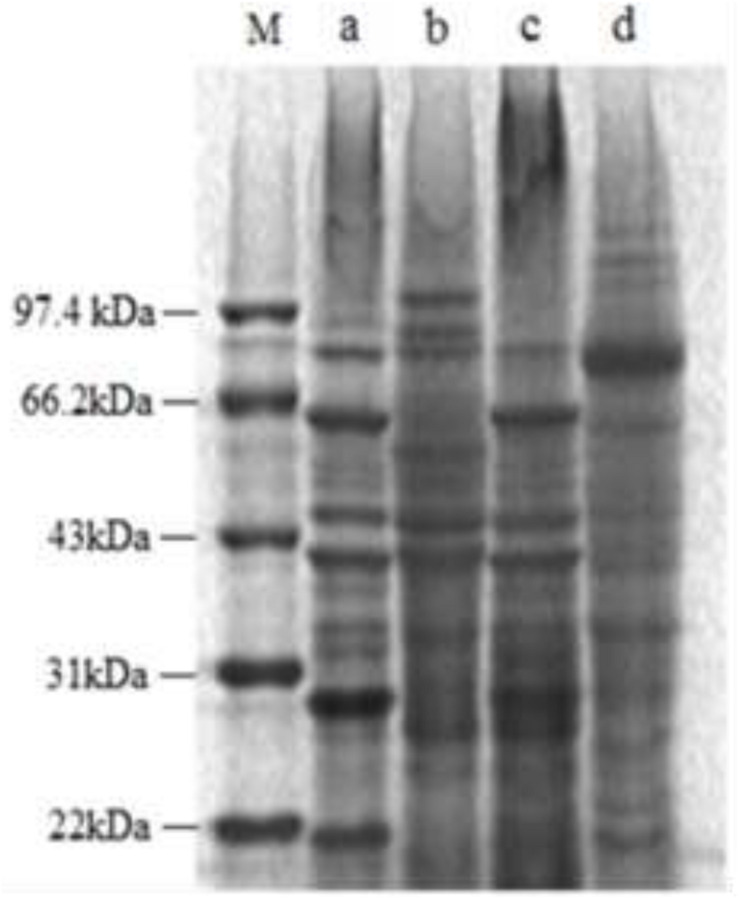
SDS-PAGE electropherogram of the two indicator bacteria, the molecular marker (M), the *E. coli* used as control (a), the treated *E. coli* (b), the *S. aureus* used as control (c), the treated *S. aureus* (d).

Figure 7 shows that some typical bands of the treated *E. coli* in SDS-PAGE electropherogram obviously disappeared and faded, and some new protein bands also appeared. For the treated *S. aureus*, most of the typical bands disappeared and faded, and an obvious new band appeared at about 70 kb. The above result illustrated that lacidophilin significantly affected the growth and the protein content of the indicator bacteria.

### Effect of Lacidophilin on Intracellular Enzyme (SOD, POD, and CAT) Activity and MDA Content

For the two treated indicator bacteria, the contents of MDA in the cells were both higher than those of the controls, and the activities of intracellular enzymes (SOD, CAT, and POD) also exceeded those of the controls significantly. The increase of MDA contents indicated that the damage to the cell increased. The increase of the activity of SOD, CAT, and POD inferred that the lipid peroxidation of the cytomembrane increased.

## Discussion

The negative control (MRS medium) had no antibacterial activity, whereas the antibacterial activity of lacidophilin and the positive control had no significant difference (*P* > 0.05), which indicated a potential application of lacidophilin as a food preservative. The heat treatment indicated that lacidophilin had relatively high thermal stability, as 90% of the bactericidal activity was retained with heat treatment at 65°C for 30 min. Thus, it could be used as a preservative in pasteurized food (Thirumurugan et al., 2016). This was similar to the previous report about the thermal stability of KF1 bacteriocin (Zahid, 2015). The result of trypsin treatment indicated that lacidophilin might be a protein or peptide that could be completely hydrolyzed by trypsin (Lv et al., 2018). The amylase treatment illustrated that the protein or peptide might contain some glycosidic bonds, as amylase mainly hydrolyzes glycosidic bonds. Certain concentration of metal ions weakened the antibacterial activity of lacidophilin (Figure 3). Previous research also indicated that divalent cations were antagonists of the bacteriocin (Vloten, 2015). Thus, it was speculated that certain concentration of metal ions might reduce the antibacterial activity of lacidophilin by changing its spatial conformation, which was associated with antimicrobial activity.

The growth curves of the treated *S. aureus* and *E. coli* lagged significantly behind the controls (Figure 4), which illustrated that the bacteriocin inhibited the dynamic growth of two indicator bacteria greatly. Common lacidophilin, such as nisin, only has an antibacterial effect on Gram-positive bacteria and has no antibacterial effect on Gram-negative bacteria (Jeong and Ha, 2018), which might ascribe the amyloid formation between the lipopolysaccharide of Gram-negative bacteria cell wall and the antibacterial peptide (Wang et al., 2014). However, the lacidophilin in this study had high antibacterial activity against both Gram-positive and Gram-negative bacteria, which further illustrated its potential use as a food preservative.

Propionyl iodide is a nucleic acid staining agent that cannot penetrate the entire cytomembrane, but can penetrate the damaged cytomembrane to stain the DNA. When PI crossed the destroyed cytomembrane and bound to DNA, the cell emitted red fluorescence (Boyd et al., 2008). FDA could pass through the cytomembrane, and the living cells with FDA staining could emit yellow-green fluorescence (Boyd et al., 2008). Therefore, the result of Figure 5 indicated that lacidophilin destroyed the cytomembrane of the bacteria and increased the cytomembrane permeability. Previous study had also suggested that the bacteriocins of bifidocin A produced from *B. animalis* played a bacteriostatic role by destroying the cytomembrane of *Listeria monocytogenes* (Liu et al., 2017).

Electrolyte, nucleic acid, and proteins are very important for the life of the indicator bacteria. The leakage of electrolyte, nucleic acid, and proteins further illustrated that the cytomembrane of the bacteria was damaged (Shen et al., 2015; Cai et al., 2019), resulting in the loss of the barrier function of the cytomembrane. The leakage might attribute to the formation of the selective pores in the bacteria cytomembrane (Liu et al., 2017), and the loss of the key cell contents affected the normal life of the cells, thereby inhibiting the growth of the bacteria.

Phosphorus is an important nutrient required for key biological reactions of cell life, and it is a crucial component of the cytomembrane (Razzaque, 2011). The metabolic capacity of the cells could be confirmed by detecting phosphorus consumption in the cultivation process (Tian and Wang, 2014). For the treated bacteria, the content of phosphorus in the inoculum decreased slower than the control, which indicated that lacidophilin inhibited the phosphorus consumption of the indicator bacteria. Decrease of phosphorus consumption further illustrated that lacidophilin destroyed the metabolic capacity of the cell, inhibited the formation of the cytomembrane, and increased the permeability of the cytomembrane (Wang et al., 2020).

Proteins are critical parts of bacteria cells, and they are closely related to the metabolism of bacteria cells. Disappearance and fade of typical bands of the cell proteins in SDS-PAGE (Figure 7) indicate that lacidophilin had a significant effect on protein and the protein contents of the indicator bacteria. The SDS-PAGE result further suggested that the cytomembrane was destroyed and the growth of the bacteria was inhibited, resulting in the fade or disappearance of typical bands of cell proteins (Cai et al., 2019). In addition, the result also corroborates that lacidophilin damaged the cytomembrane of the indicator bacteria, leading to the leakage of major cell components, which seriously affected the synthesis of protein (Meng et al., 2016). The appearance of new protein bands might be that the bacteriocin changed the metabolism of bacterial cells, resulting in the production of new proteins.

**FIGURE 8:**
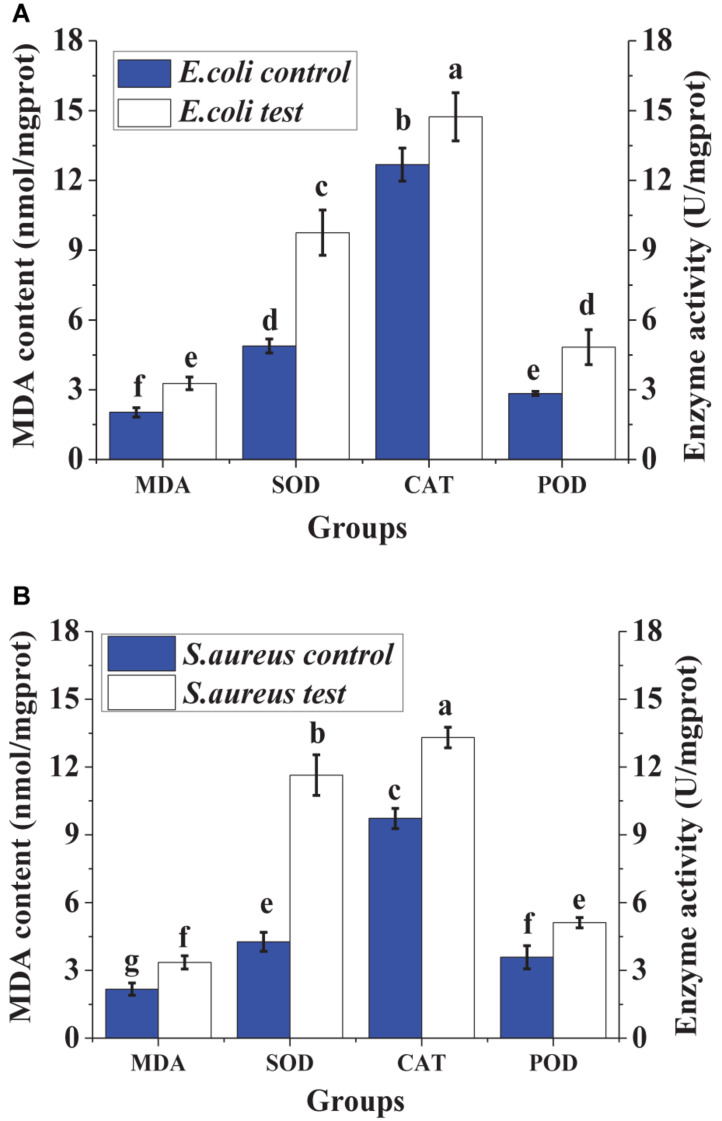
Effect of lacidophilin on intracellular enzymes (SOD, POD and CAT) activity and MDA content of the two indicator bacteria, the *S. aureus* **(A)** and the *E. coli* **(B)**.

Malondialdehyde is the crucial lipid peroxidation product, which can be used to monitor the lipid peroxidation level of the cytomembrane (Jia et al., 2014). The increase of MDA content illustrated the increase of lipid peroxidation of the cytomembrane, resulting in the damage of the cytomembrane (Yun et al., 2013). The cellular enzymes including SOD, POD, and CAT can be used to detect the cell defense response against external stress (Yun et al., 2013). In order to protect itself from external injury, the activities of the enzymes including SOD, CAT, and POD in the cell were initiated, resulting in an increase of reactive oxygen removal and a reduction of oxidative damage (Jia et al., 2014). Therefore, the increase of the activities of SOD, CAT, and POD in the cell (Figure 8) illustrated that the cell was injured by lacidophilin. Thus, the result indicated that lacidophilin caused cytomembrane lipid peroxidation and cell oxidative damage simultaneously.

## Conclusion

In this paper, the antibacterial activity of *L. pentosus* lacidophilin against *S. aureus* and *E. coli* was studied, and the antibacterial mechanism was further explored. The results showed that lacidophilin might be a polypeptide or protein, which had significant antibacterial activity against *S. aureus* and *E. coli*. Lacidophilin had strong thermal stability, so it might be used in pasteurization processing of foods. The antibacterial activity was decreased by metal ions; therefore, the influence of metal ions should be avoided during food processing using lacidophilin as preservative. Studies on antibacterial mechanism showed that lacidophilin mainly broke the integrity of the bacteria cytomembrane and increased the permeability of the cytomembrane by melting the cytomembrane into holes, further leading to the leakage of electrolytes, nucleic acid, and proteins. In addition, it restrained phosphorus metabolism, inhibited the growth of the bacteria, and caused changes of some proteins. Moreover, lacidophilin injured the bacteria cell, which increased cytomembrane lipid peroxidation and cell oxidative damage, resulting in the increase of MDA content and activities of SOD, CAT, and POD in the cell. Consequently, lacidophilin has great potential for application as natural preservatives in foods, owing to their high antibacterial activity against both Gram-positive and Gram-negative foodborne pathogens and thermal stability.

## Data Availability Statement

The raw data supporting the conclusions of this article will be made available by the authors, without undue reservation.

## Author Contributions

YZ designed the study and drafted the original manuscript. SZ was responsible for the method, data acquisition, curation, and analysis. All authors contributed to the article and approved the submitted version.

## Conflict of Interest

The authors declare that the research was conducted in the absence of any commercial or financial relationships that could be construed as a potential conflict of interest.
